# Research progress on the chemical composition of galli gigeriae endothelium corneum

**DOI:** 10.3389/fchem.2025.1644192

**Published:** 2025-07-23

**Authors:** Guo-Lian Gan, Hongxin Zhou, Zhen-Bin Lin, Xiang Li, Jing-Jin Lin, Li Zhang

**Affiliations:** ^1^Guangdong Provincial Hospital of Chinese Medicine, The Second Affiliated Hospital of Guangzhou University of Chinese Medicine, Guangzhou, Guangdong, China; ^2^ Guangdong Pharmaceutical University, Guangzhou, Guangdong, China; ^3^ Guizhou Province Zunyi City Suiyang County Hospital of Traditional Chinese Medicine, Zunyi, Guizhou, China; ^4^ Guangdong Women and Children Hospital, Guangzhou, Guangdong, China

**Keywords:** galli gigeriae endothelium corneum, traditional Chinese medicine, pharmacological activities, bioactive components, chemical constituents

## Abstract

Galli Gigeriae Endothelium Corneum (GGEC), commonly known as “Ji Nei Jin” in traditional Chinese medicine (TCM), is derived from the dried inner lining of the chicken gizzard. It has been widely used for centuries in China for treating indigestion, enuresis, gallstones, and urinary calculi. Recent studies have revealed that GGEC contains a variety of bioactive constituents, including proteins, amino acids, peptides, enzymes, and trace elements, which contribute to its diverse pharmacological activities. Modern pharmacological investigations have demonstrated its efficacy in promoting gastrointestinal motility, enhancing digestive enzyme activity, regulating glucose metabolism, dissolving stones, and exerting anti-inflammatory and hepatoprotective effects. Moreover, clinical and experimental research has supported its potential as an adjunct in treating metabolic and digestive disorders. Despite these promising findings, limitations such as unclear mechanisms of action, lack of standardized preparations, and insufficient clinical trials hinder its broader application. This review aims to summarize the traditional uses, chemical constituents, pharmacological activities, and current research progress of GGEC, and to provide a reference for future studies and clinical utilization.

## 1 Introduction

### 1.1 Source and processing

Galli Gigeriae Endothelium Corneum (GGEC), commonly known in Traditional Chinese Medicine (TCM) as “Ji Nei Jin,” is the dried inner lining of the gizzard (a muscular portion of the stomach) of Gallus *gallus domesticus* Brisson. This material presents as a golden-yellow keratinous layer. GGEC is a frequently utilized medicinal component in TCM clinical practice. The preparation involves harvesting the gizzard from a freshly slaughtered chicken, immediately separating the inner lining, followed by washing and drying (Pharmacopoeia, 2020). GGEC is sourced from various regions across China, where it is abundantly available. Common processing methods include stir-frying, sand-blanching, and vinegar-roasting. Some are directly dried and used as medicine, while others are stir-fried with auxiliary ingredients. Vinegar-roasted GGEC preparations are predominantly found in southern China, whereas stir-fried preparations are more common in the north (Fan et al., 2021).

### 1.2 Traditional functions and indications

The earliest record of GGEC appears in the “Shennong Bencao Jing” (Shennong’s Materia Medica Classic), where it was classified as a top-grade medicinal (Zhi, 2022). In TCM theory, GGEC is characterized by a sweet taste and neutral nature, with therapeutic actions attributed to the Spleen, Stomach, Small Intestine, and Bladder meridians. Its traditional functions include strengthening the Stomach to improve digestion, acting as an astringent to resolve stagnation, and generally promoting digestive processes (Chen et al., 2023). Raw (unprocessed) material is cleaned, sun-dried, and used directly to preserve its natural components. Stir-frying over low heat until browning occurs invigorates the spleen, stimulates appetite, and facilitates digestion by alleviating food stagnation. Sand-blanching is performed by heating with hot sand until the material puffs up and becomes crispy, improving the extraction efficiency of active constituents. Vinegar-roasting involves stir-frying or decocting with rice vinegar to guide the effects toward the liver meridian and enhance functions like dissolving calculi and astringing essence. Auxiliary material-frying includes processing with Zao Xin Tu (earth from the stove core) to reinforce digestive benefits, clam shell powder to strengthen stone-dissolving properties for treating biliary or renal calculi, and salt or talc powder, used in certain regions and scientifically supported by enzyme activity studies. Consequently, GGEC is indicated for conditions such as indigestion, vomiting, diarrhea, infantile malnutrition, enuresis, nocturnal emissions, urolithiasis (historically termed “stone gonorrhea” in some texts), and pain associated with gallbladder distension (Lu et al., 2006).

### 1.3 Commercial formulations containing GGEC

Several compound formulations incorporating GGEC are commercially available, primarily addressing digestive and pediatric conditions. Examples include:

Compound GGEC Tablets: These tablets are formulated to invigorate the Spleen, stimulate appetite, and aid digestion by reducing food accumulation. They are indicated for symptoms such as food stagnation, abdominal bloating, vomiting, and diarrhea attributed to Spleen-Stomach disharmony. (Primary ingredients: GGEC, *Massa Medicata Fermentata* (Liu Shen Qu)).

Pediatric Compound GGEC Powder: This powder is designed to invigorate the Spleen, stimulate appetite, and promote the digestion and resolution of food accumulation in children. It is used for pediatric cases of food stagnation and abdominal bloating resulting from Spleen-Stomach disharmony, as well as associated vomiting and diarrhea. (Primary ingredients: GGEC, *Massa Medicata Fermentata* (Liu Shen Qu). Excipients: White Sugar, Starch).

Huaji Tablets: These tablets primarily function to promote digestion. They are indicated for children experiencing Spleen-Stomach disharmony, characterized by loss of appetite (including for milk in infants), abdominal masses or distension, fatigue, sallow complexion, and poor appetite. (Ingredients: *Crataegus pinnatifida* (Hawthorn, stir-fried), *Hordeum vulgare* (Malt, stir-fried), *Massa Medicata Fermentata* (Liu Shen Qu, stir-fried with bran), *Areca catechu* (Betel Nut, stir-fried), GGEC (stir-fried), *Pharbitis nil* (Morning Glory Seed, stir-fried)).

Jianpi Zhiyi Tablets: The tablets are formulated to invigorate the Spleen and Stomach, reduce enuresis, and alleviate stagnation. They are used for treating enuresis in children associated with Spleen-Stomach disharmony. (Ingredients: Chicken intestine, GGEC. Excipients: Starch, ethanol, magnesium stearate, film coating agent).

Gandanqing Capsules: These capsules aim to clear Heat and Dampness, promote bile flow, and facilitate the removal of stones. They are indicated for cholecystitis and cholelithiasis attributed to Damp-Heat in the Liver and Gallbladder. (Ingredients: *Desmodium styracifolium* (Pig Mane Grass/Guang Jin Qian Cao), *Lysimachia christinae* (Golden Grass/Jin Qian Cao), *Gentiana scabra*, *Rheum palmatum* (Rhubarb), *Coptis chinensis*, *Corydalis yanhusuo*, GGEC, *Ochra Haematitum* (Ochre), *Evodia rutaecarpa*, Micropowder Silica Gel, Crosslinked Polyvinylpyrrolidone).

Gallstone Tablets: These tablets are designed to soothe the Liver, promote bile flow, enhance Qi circulation, and relieve pain. They are used for gallbladder stones and intrahepatic bile duct stones presenting with Qi stagnation syndrome. (Main ingredients: Oxgall, Saltpeter, GGEC (stir-fried), *Fructus Aurantii*, *Cyperus rotundus*, *Aucklandiae radix*, *C. yanhusuo*, *C. chinensis* Franch., *Atractylodes macrocephala* Koidz., *Tetradium ruticarpum*, *Alpinia officinarum* Hance, *C. pinnatifida* (Hawthorn), etc.).

Bao’er Ning Granules: The granules are formulated to tonify Qi, strengthen the exterior (Wei Qi), reinforce the Middle Jiao (Spleen and Stomach), and awaken the Spleen. They are indicated for fatigue, drowsiness, sallow complexion, muscle thinness, restlessness, spontaneous sweating due to exterior deficiency, and susceptibility to common colds, all arising from Spleen and Lung Qi deficiency. (Ingredients: *Astragalus propinquus* (Huangqi, roasted), *A. macrocephala* (Bai Zhu, stir-fried), *Saposhnikovia divaricata* (Fang Feng/Windproof Root), *Phragmites communis* (Reed Root), GGEC, *Poria cocos*, *Dioscorea polystachya* (Yam, stir-fried)).

## 2 Common analytical methods for GGEC

### 2.1 High performance liquid chromatography (HPLC)

Wang et al. isolated β-glucosidase from GGEC and employed HPLC to assess its hydrolytic activity on soy isoflavones. Their findings indicated that the enzyme effectively hydrolyzed soy isoflavone glycosides (at least six isomers) with a conversion rate of 99%. This study provided a basis for further investigation into the hydrolytic characteristics of GGEC β-glucosidase (Wang et al., 2010).

Liang, Zhang, and colleagues developed a pre-column derivatization Reverse Phase HPLC (RP-HPLC) method for the determination of 16 amino acids in GGEC. The method was reported to be simple, precise, and reproducible, demonstrating its suitability for quantifying hydrolyzed amino acids in GGEC samples (Liang et al., 2014). Xiu Yanfeng et al. used HPLC to detect the flavonoid substances (genistin, glycitin, puerarin, genistein, daidzein, and puerarin) in GGEC. They established a quantitative detection method for GGEC components and a quality evaluation method for GGEC, further enabling effective judgment of whether GGEC samples meet quality standards (Xiu et al., 2022).

### 2.2 Titration and UV spectrophotometry

Cai investigated the polysaccharide and mucopolysaccharide content in different processed forms of GGEC. Mucopolysaccharides were extracted using alkaline extraction and quantified via UV spectrophotometry, while amylase activity was determined by titration. The study revealed that domesticated chicken GGEC possessed a higher mucopolysaccharide content compared to that from white feather chickens. Conversely, GGEC from white feather chickens exhibited higher amylase and gastric pepsin activities (Cai et al., 2015).

### 2.3 Atomic absorption spectroscopy

Hu Tinghong, Hu Jiuhong, Zhou Bing, and others from the School of Life Science and Engineering, Southwest Jiaotong University, used atomic absorption spectroscopy to measure the content of five metal elements (Mg, Fe, Mn, Zn, Cu) in GGEC. The results showed that the GGEC samples contained relatively rich levels of Mg, Fe, Mn, Zn, and Cu, with recovery rates ranging from 95.27% to 104.47% and RSD values less than 3.53%.

At the Testing Center of Jilin Agricultural University, Li Zehong, Chen Dan, and Li Zhenhua used atomic absorption spectroscopy to measure the nutritional elements in GGEC. The results showed that the contents of the nutritional elements potassium, calcium, magnesium, copper, zinc, iron, and manganese in GGEC were 369.50, 120.56, 470.77, 31.89, 32.42, 559.64, and 8.11 mg/kg, respectively. This method established an analytical approach for metal elements in GGEC, which is simple to operate, precise, and reliable, making it suitable for measuring metal elements in GGEC (Hu et al., 2011).

### 2.4 Electrophoresis detection method

Xu, Zhang, and colleagues from Fujian Medical University utilized high performance capillary electrophoresis (HPCE) for the electrophoretic analysis of acidic, neutral, and alkaline proteins in GGEC and its potential adulterants. The study demonstrated that HPCE profiles and the absorption peaks of GGEC extracts exhibited significant differences across three distinct extraction solutions, facilitating their differentiation. This suggests HPCE can serve as an identification technique to distinguish authentic GGEC from adulterated samples (Xu et al., 2007).

### 2.5 Gas chromatography-mass spectrometry (GS-MS) analysis method

By combining the advantages of gas chromatography (GC) and mass spectrometry (MS), both quantitative and qualitative capabilities are enhanced. Qingping Xiong et al. utilized GC-MS analysis to study the characterization and biological activity of a novel purified polysaccharide extracted from GGEC. The experiments showed that PECGp has a molecular weight of 96 kDa. Measurements of cardiac function indicated that PECGp significantly reduced ST-segment elevation, prevented myocardial morphological changes, reversed abnormal hemodynamic and blood rheology parameters, and corrected disrupted levels of superoxide dismutase, nitric oxide synthase, and nitric oxide. The PECGp extracted from GGEC can be considered a potential candidate for the development of new cardioprotective agents (Xiong et al., 2015).

### 2.6 UPLC-Q-TOF-MS

Fan et al. analyzed the chemical constituents of an aqueous extract of GGEC using UPLC-Q-TOF-MS. Component identification was based on a comparison of retention times, quasi-molecular ions (from primary mass spectra), and characteristic fragment ions (from secondary mass spectra) with those of reference standards and data from existing literature. This approach led to the identification of 10 nucleoside compounds and 3 amino acid components in the aqueous extract. Notably, this study reported the first identification of these 10 nucleosides in GGEC aqueous extracts. Furthermore, an HPLC fingerprinting method and a quantification method for 7 of these nucleoside components were subsequently established based on these findings (Fan et al., 2023).

## 3 Chemical compositions and biological activities of GGEC

The nutritional components of GGEC mainly include protein and peptides, amino acids, polysaccharides, metal elements, and flavonoids.

### 3.1 Proteins and peptides

GGEC is rich in proteins and peptides. By using alkaline water extraction, the yield of GGEC extract can reach 42.37%. Through analysis of the total amino acid content, it was found that the protein content accounts for about 60% (Liu and Ren, 2004).

Chien Hsiang Ni et al. analyzed the soluble proteins in GGEC and found that the main proteins were gastric antral mucosal protein, zinc finger protein, and serum albumin precursor. Among them, gastric antral mucosal protein was the most abundant protein in GGEC (21.94%); This protein positively regulates activities related to cell proliferation and promotes healing in gastric injury by promoting recovery and proliferation after injury (Ni et al., 2018).

Li, Shanshan et al. identified a new pentapeptide Leu-Asn-Leu-Tyr-Pro from GGEC and found that this novel peptide activates the aryl hydrocarbon receptor (AhR), which can inhibit Src kinase, increase tight junction protein levels, and downregulate the expression of inflammatory cytokines, protect the intestinal barrier, and ultimately alleviate dextran sulfate sodium (DSS)- induced colitis (Li et al., 2020).

Guo, RX et al. isolated and purified active peptide B1 with the ability to repair gastric mucosal damage from GGEC, and demonstrated its significant activity in repairing gastric mucosal damage. The component consists of six peptide segments, including DYPELS(Asp-Tyr-Pro-Glu-Leu-Ser), LPPEH (Leu-Pro-Leu-Glu-His), SFYYGK(Ser-Phe-Tyr-Tyr-Tyr-Gly-Lys), DDDGVGF(Asp-Asp-Asp-Gly-Gly-Val-Gly-Phe), VVLPPR(Val-Val-Leu-Pro-Arg) and LPYPR(Leu-Pro-Tyr-Pro-Arg). Peptide segments LPPLEH and LPYPR exhibit excellent scratch repair capabilities (Guo et al., 2024).

### 3.2 Amino acids

The total content of amino acids in dried GGEC was determined to be as high as 86.92% using an automatic amino acid analyzer, with essential amino acids accounting for 30.3%. Among the 14 detected amino acids, proline had the highest content, reaching over 3 mg per gram of medicinal material, followed by lysine, methionine, glycine, leucine, and phenylalanine, all at around 1 mg/g (Fan et al., 2021).

### 3.3 Nucleoside

Nucleoside components are the basic building blocks for maintaining the activities of living organisms, with biological activities such as anti-tumor, antiviral, antibacterial, anti-inflammatory, and immune regulation (Ding et al., 2018). Fan Jia et al. identified 10 types of nucleoside components from GGEC for the first time, including uracil, cytidine, hypoxanthine, uridine, thymine, adenine, inosine, guanosine, thymine, and adenosine (Fan et al., 2023).

### 3.4 Polysaccharides

**FIGURE 1:**
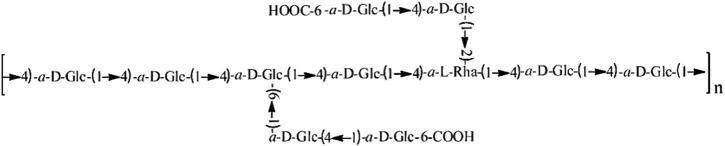
Structure diagram of PECGp (a novel purified polysaccharide from GGEC).

The study investigated that characterization *in vivo* of a novel purified polysaccharide (Polysaccharides from E. corneum gigeriae galli, hereinafter referred to as PECG) from GGEC showed that PECG is composed of rhamnose, glucose, fucose, mannose, and galactose (Xiong et al., 2014); polysaccharides from Endothelium corneum gigeriae galli (PECGp) was thoroughly characterized by Xiong et al. using gas chromatography-mass spectrometry (GC-MS). This study found that PECGp had a 96 kDa molecular weight, and the backbone chains were composed of α-D-Glc and α-L-Rha linked by (1→4) glycosidic bonds. The molecular structure is shown in Figure 1. By evaluating its cardioprotective activity in a myocardial ischemia model, it has been confirmed that PECGp have significant *in vivo* cardioprotective effects. In addition, PECGp may exert their cardioprotective effects by enhancing the endogenous antioxidant defense system, maintaining the antioxidant balance of myocardial cells, increasing levels of nitric oxide (NO) and nitric oxide synthase (NOS), and reverse abnormal hemodynamic and hemorheological parameters. The results suggested that PECGp could be considered as a potential candidate for developing novel cardioprotective agents (Xiong et al., 2015).

### 3.5 Metal elements

GGEC contains abundant metal elements. Using flame atomic absorption spectroscopy, the highest contents of Fe, Mg, Cu, Zn, and Mn in GGEC samples can reach 717.6 μg/g, 246.6 μg/g, 18.7 μg/g, 38.5 μg/g, and 67.4 μg/g, respectively. These elements are components of many important enzymes in the body and participate in various physiological functions. The abundance of these elements in GGEC may be related to its functions of promoting digestion, astringency, and detoxification (Hu et al., 2011).

### 3.6 Flavonoids and bile acid components

**FIGURE 2:**
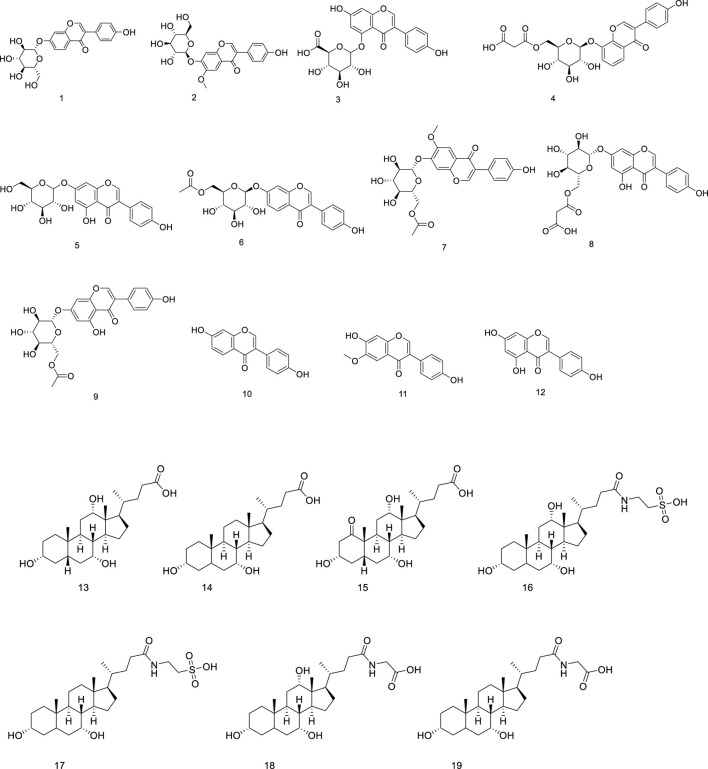
Structural formula of Flavonoids and bile acid in GGEC.

Li, Shanshan et al. found that GGEC ethyl acetate extract (EAE) can reduce the release of pro-inflammatory cytokines, indicating its potential beneficial role in intestinal epithelial barrier function. A total of 19 compounds were identified by HPLC-QTOF-MS/MS, as shown in Table 1, including 12 soy isoflavones (daidzin, daidzein, genistein O-glucuronide, 6″- O-malonyl daidzein, genistein, 6″- O-acetyl genistein, malonyl genistein, 6″- O-acetyl genistein, daidzein, genistein and genistein), and 7 bile acids (cholic acid, chenodeoxycholic acid, Oxocholic acid, taurocholic acid, taurodeoxycholic acid, glycocholic acid and glycodeoxycholic acid), their chemical structure is shown in Figure 2 (Li S. et al., 2021).

**TABLE 1:** Chemical composition of GGEC (CAS: Chemical abstracts service).

NO.	Formula	Identification	CAS number
1	C21H20O9	Daidzin	552-66-9
2	C22H22O10	Glycitin	40246-10-4
3	C21H18O11	Genistein-*O*-glucuronide	1373230-82-0
4	C24H22O12	6″-*O*-Malonyldaidzin	124590-31-4
5	C21H20O10	Genistin	529-59-9
6	C23H22O10	6″-*O*-Acetyldaidzin	71385-83-6
7	C24H24O11	6″-*O*-Acetylglycitin	134859-96-4
8	C24H22O13	6″-Malnoylgenistin	51011-05-3
9	C23H22O11	6″-*O*-Acetylgenistin	73566-30-0
10	C15H10O4	Daidzein	486-66-8
11	C16H12O5	Glycitein	40957-83-3
12	C15H10O5	Genistein	446-72-0
13	C26H45NO7S	Taurocholic acid	81-24-3
14	C26H43NO6	Glycocholic acid	475-31-0
15	C24H38O5	Oxocholic acid	2304-89-4
16	C26H45NO6S	Taurochenodeoxycholic acid	516-35-8
17	C24H40O5	Cholic acid	81-25-4
18	C26H43NO5	Glycochenodeoxycholic acid	640-79-9
19	C24H40O4	Chenodeoxycholic acid	474-25-9

Liu Qinghao established an HPLC method for determining the content of six flavonoids: daidzin, glycitin, genistin, daidzein, glycitein, and genistein. Using them as quantitative indicators. A comparative analysis was conducted on their levels in raw and processed Galli Gigeriae Endothelium Corneum (GGEC). The average contents in raw samples were 1.10, 0.22, 1.33, 0.27, 0.77, and 0.62 mg/g, respectively, while in processed samples they were 0.94, 0.20, 1.12, 0.77, 2.32, and 1.75 mg/g. These flavonoids are recognized as bioactive constituents in many traditional Chinese medicines. Specifically, daidzin exhibits anti-osteoporotic properties; daidzein has hypoglycemic and antibacterial activities; glycitin shows α-glucosidase inhibition and blood glucose-lowering effects; glycitein demonstrates antioxidant activity; and both genistin and genistein possess multiple pharmacological functions, including antioxidant, anti-tumor, lipid-lowering, anti-hepatic fibrosis, and anti-estrogenic effects (Liu et al., 2023).

## 4 Pharmacological activities of GGEC

### 4.1 Effects on the digestive system

Li Feiyan et al. examined the effects of raw GGEC (aqueous decoction prepared according to pharmacopoeial methods) and its processed forms on gastric juice secretion and pepsin activity in rats. Their methodology involved continuous gastric gavage, fasting, dissection, ligation, and gastric fluid collection. The results indicated that, compared to the control group, gastric juice volume was significantly increased in all GGEC-treated groups (P < 0.05). Furthermore, processed forms of GGEC led to varying degrees of increased gastric juice volume compared to the pharmacopoeia-defined raw product. While the raw product group did not significantly alter gastric pepsin activity relative to the control, all processed groups significantly enhanced gastric pepsin activity (P < 0.05). Additionally, all GGEC treatment groups demonstrated a significant increase in the excretion of gastric pepsin in rats (Li et al., 2008).

GGEC aqueous extracts at low, medium, and high doses (1, 2, and 4 g raw drug/kg) were administered by continuous gastric gavage to rats with functional dyspepsia (FD) for 7 days. The results showed that these extracts reduced the expression level of endothelial nitric oxide synthase (eNOS) protein in gastric tissue. Cao Feng et al. found that the improvement of FD was related to the expression of AQP4 and eNOS proteins in gastric tissue, suggesting that the pathogenesis of FD is associated with the expression of AQP4 and eNOS. The medium and high doses (2 and 4 g raw drug/kg) also significantly increased serum levels of gastrin and motilin in FD rats, upregulated the expression of aquaporin 4 in gastric tissue, and thus improved the gastrointestinal function in the FD rat model (Shen et al., 2019).

Chi Yusen et al. investigated the effects of GGEC extract on intestinal health in mice, utilizing the charcoal meal intestinal propulsion method to assess intestinal transit rates. Their findings revealed that GGEC extract exerted differential effects on murine intestinal motility depending on the dosage. No significant differences were observed between the low and medium-dose groups (P > 0.05); however, the high-dose group exhibited highly significant improvements compared to the control group (P < 0.01). The GGEC-treated groups also showed a shortened time to first defecation, an increased number of fecal pellets, and increased fecal weight, indicating that GGEC enhances small intestinal motility and alleviates constipation in a dose-dependent manner (Chi et al., 1999).

### 4.2 Effects on the circulatory system

Studies by Ma et al. (2003), Guo et al. (2000), and other researchers, using rabbits as experimental models, analyzed blood glucose, blood lipids, and hemorheological parameters from samples obtained via the marginal ear artery. These investigations demonstrated that the combined administration of GGEC and hawthorn reduced blood glucose and lipid levels in hyperlipidemic rabbit models, along with a decrease in liver and mesenteric weight. These results suggest that GGEC, particularly in combination with hawthorn, possesses hypoglycemic and lipid-lowering properties, and can reduce fat accumulation in the liver and mesentery. Moreover, GGEC exhibited inhibitory effects on the coagulation system and improved blood rheology, demonstrating some preventive activity against the development of atherosclerosis.

Jiang Changxing et al. explored the effects of GGEC-derived polysaccharides on hyperlipidemia in rat models, focusing on changes in blood lipid profiles. Their results showed that in the polysaccharide-treated group, blood lipid and hemorheological indices approached normal physiological levels, and oxidative stress responses were attenuated. This suggests that GGEC polysaccharides can effectively ameliorate lipid metabolism disorders, thereby contributing to the normalization of metabolic parameters (Jiang et al., 2012).

### 4.3 Effects on the urinary system

Nan Wang et al. demonstrated that GGEC extract can inhibit the nucleation and growth of calcium oxalate kidney stones. This effect was attributed to its ability to lower urinary levels of uric acid, oxalate, calcium, creatinine, urea nitrogen, and phosphate, while concurrently increasing magnesium content and urinary excretion. Additionally, the extract was found to protect kidneys from damage by reducing serum creatinine, urea nitrogen, and uric acid levels, and by enhancing the activity of superoxide dismutase (SOD) (Wang et al., 2019).

### 4.4 Effects on the endocrine system

Liu Yuanxin established a rat model of mammary gland hyperplasia using benzophenone estradiol and progesterone, observing the morphological changes of the rats’ mammary glands. The results showed that the GGEC group experienced some relief in mammary gland morphology (Liu, 2016).

Hu Jianping et al. investigated the effects of raw GGEC on mammary gland hyperplasia in rats with liver qi stagnation and spleen deficiency. *In vitro* experiments demonstrated that raw GGEC effectively improved the symptoms of mammary gland hyperplasia in rats. Furthermore, when combined with Xiaoyao Powder, a stronger therapeutic effect was observed (Hu et al., 2015).

### 4.5 Effects on the reproductive system

Inhibition of uterine fibroid growth. Uterine fibroids are classified in traditional Chinese medicine as “symptoms and masses,” resulting from qi stagnation and blood stasis, which block the uterus. Wang Xiaoping et al. studied the treatment of intramural fibroids, using raw GGEC in combination with Gui Zhi Fu Ling Capsule as the treatment group, and Gui Zhi Fu Ling Capsule alone as the control group. Fibroid volume changes and blood rheology were used as observation indicators. The results showed that the treatment group had an overall effective rate of 93.3%. This indicates that raw GGEC can effectively inhibit the growth of uterine fibroids, thus providing a significant therapeutic effect for the treatment of uterine fibroid-related conditions (Wang and Cui, 2013).

### 4.6 Other effects

Li Ye et al. discovered that GGEC extract exerts anti-inflammatory and anti-fibrotic effects by inducing autophagy in alveolar macrophages (Li Y. et al., 2021). Application of GGEC in the treatment of oral mucosal repair. Oral ulcers can lead to difficulty in eating, resulting in a decline in the patient’s quality of life. GGEC has proven to be effective in treating oral ulcers. A study found that treatment with GGEC and vitamin B2 in 38 children with oral thrush achieved a 100% cure rate. For chemotherapy-induced oral ulcers, GGEC powder showed significant therapeutic effects (Wang et al., 2017).

Application of GGEC in improving chronic renal failure. Research showed that the treatment rate was 26.92% for the group treated with the basic formula for renal failure combined with GGEC, compared to 14.29% for the group treated with the basic formula alone. This suggests that the compound containing GGEC has a positive effect on improving chronic kidney disease, laying the foundation for the development of new drugs for chronic renal failure (Zhang, 2015).

Application of GGEC in regulating cardiac function. Polysaccharides extracted from GGEC significantly reduced ST-segment elevation, prevented myocardial morphological changes, and regulated the enzyme levels of superoxide dismutase, nitric oxide synthase, and lactate dehydrogenase. These findings provide a basis for the development of cardioprotective agents. Subsequent research revealed that ammonium chloride in GGEC can promote the excretion of strontium, although the mechanism remains unclear and warrants further investigation (Luo and Chen, 2000).

## 5 Conclusion and prospect

GGEC, a commonly used traditional Chinese medicine derived from the inner lining of the chicken gizzard, demonstrates a rich pharmacological profile and diverse therapeutic applications. Through various traditional and modern processing techniques, including stir-frying, sand-blanching, and vinegar-roasting, the bioactive components of GGEC are either preserved or enhanced, aligning with its classical functions of promoting digestion, resolving stagnation, and addressing a range of pediatric and gastrointestinal disorders.

Phytochemical and analytical studies have revealed that GGEC is abundant in proteins, peptides, amino acids, polysaccharides, flavonoids, metal elements, and nucleosides, each contributing to its biological efficacy. Advanced analytical methods such as HPLC, UPLC-Q-TOF-MS, atomic absorption spectroscopy, and GC-MS have facilitated the identification and quantification of its major constituents, offering a foundation for standardization and quality control.

Modern pharmacological investigations have corroborated GGEC’s traditional claims, demonstrating its multifaceted effects on the digestive, circulatory, urinary, endocrine, reproductive, and immune systems. Notably, it shows promise in alleviating functional dyspepsia, improving lipid metabolism, preventing urolithiasis, modulating hormonal imbalances, and exerting anti-inflammatory, cardioprotective, and nephroprotective effects. These findings not only validate its long-standing use in traditional practice but also highlight its potential in developing novel therapeutic agents.

In conclusion, GGEC exemplifies the integration of traditional wisdom and modern science, offering a valuable natural product with broad clinical applications. Continued research into its mechanisms of action, bioactive constituents, and clinical efficacy will further elucidate its role in contemporary medicine and support its development as a standardized and evidence-based therapeutic resource.

While extensive research, particularly within Chinese literature, has explored the diverse pharmacological effects of GGEC extracts, in-depth studies focusing on the precise identification and characterization of its bioactive components are comparatively less common. This has resulted in an incomplete understanding of the specific molecules responsible for its therapeutic actions. In the international scientific literature, although studies have begun to characterize certain constituents, such as peptides and polysaccharides in GGEC, and investigate their respective bioactivities, comprehensive research into its overall chemical profile remains relatively limited. Consequently, the exact active ingredients and their detailed mechanisms of action in GGEC warrant more rigorous investigation. Furthermore, the development of value-added products derived from GGEC is still in its nascent stages. Therefore, a crucial direction for future research involves a more thorough elucidation of GGEC’s pharmacologically active compounds and their modes of action. Additionally, further exploration of its clinical application value, supported by well-designed clinical trials, and the subsequent development of standardized, high-value health products and pharmaceuticals derived from GGEC, represent important avenues for advancing its therapeutic potential.
